# Omics-based analysis of *Akkermansia muciniphila* cultivation in food-grade media

**DOI:** 10.20517/mrr.2024.06

**Published:** 2024-06-17

**Authors:** Sharon Y. Geerlings, Kees van der Ark, Bart Nijsse, Sjef Boeren, Mark van Loosdrecht, Clara Belzer, Willem M. de Vos

**Affiliations:** ^1^Laboratory of Microbiology, Wageningen University, Wageningen 6708 WE, the Netherlands.; ^2^Laboratory of Systems and Synthetic Biology, Wageningen University, Wageningen 6708 WE, the Netherlands.; ^3^Laboratory of Biochemistry, Wageningen University, Wageningen 6708 WE, the Netherlands.; ^4^Department of Biotechnology, Delft University of Technology, Delft 2629 HZ, the Netherlands.; ^5^Human Microbiome Research Program, Faculty of Medicine, University of Helsinki, Helsinki 00014, Finland.

**Keywords:** *A. muciniphila*, food-grade medium, human gut microbiota, industrial production

## Abstract

**Background and Aim:** Over the past years, the gut microbiota and its correlation to health and disease has been studied extensively. In terms of beneficial microbes, an increased interest in *Akkermansia muciniphila* (*A. muciniphila*) has been observed since its discovery. Direct evidence for the role of *A. muciniphila* in host health has been provided in both mice and human studies. However, for human interventions with *A. muciniphila* cells, industrial-scale fermentations are needed, and hence, the used cultivation media should be free of animal-derived components, food-grade, non-allergenic and allow for efficient growth to high densities to provide cost-effective production platforms. In this study, we assessed the growth and performance of *A. muciniphila* in batch bioreactors using newly developed plant-based media.

**Methods:** The bioreactors were supplemented with varying carbon sources, including different ratios of N-acetylglucosamine (GlcNAc) and glucose. We monitored the growth of *A. muciniphila* in the plant-based medium using optical density (OD600) measurements and microscopy. In addition, we used a combination of biochemical analysis as well as transcriptional and proteomics analysis to gain detailed insight into the physiology.

**Results:** Comparisons between growth on these media and that on mucin revealed differences at both transcriptome and proteome levels, including differences in the expression of glycosyltransferases, signaling proteins, and stress response. Furthermore, elongated cells and higher OD600 values were observed using the plant-based media as compared to cultivation media containing mucin.

**Conclusion:** These differences do not hamper growth, and therefore, our data suggest that the food-grade medium composition described here could be used to produce *A. muciniphila* with high yields for therapeutic purposes.

## INTRODUCTION

Over the past years, the gut microbiota and its correlation to health and disease have been studied extensively^[1]^. Notably, strong correlations have been made between the gut microbiota composition and diseases, such as obesity^[2,3]^, pre-diabetes^[4,5]^, type 2 diabetes, non-alcoholic fatty liver disease^[6]^ and liver cirrhosis^[7]^. Moreover, a causal involvement of gut microbiota by fecal microbiota transplantation has been demonstrated in various inflammatory and metabolic diseases^[8]^. This is increasing the interest in the development of interventions aiming to alter the gut microbiota, including those with specific gut bacteria, also termed next-generation beneficial microbes^[9-11]^.

While most gut bacteria inhabit the lumen of the colon and thrive on dietary leftovers, *Akkermansia muciniphila* (*A. muciniphila*) is an abundant gut symbiont feeding on the colonic mucosa^[12-14]^. *A. muciniphila* is a Gram-negative bacterium belonging to the Verrucomicrobia phylum, found to be present in the mucosal layer and specialized in the use of mucin as a single carbon, nitrogen, and energy source^[12,15]^. Considerable interest in *A. muciniphila* derives from human association studies that have demonstrated an inverse correlation with diabetes and obesity, as well as positive correlations with healthy metabolic status, as recently reviewed^[14]^. These results were initially found with deep metagenomic analyses, later expanded with species-targeted quantifications, and recently supported by linking thousands of *A. muciniphila* metagenomes to host characteristics^[3,16,17]^.

Direct evidence for the role of *A. muciniphila* was provided in a series of mouse models where the administration of its cells was found to prevent diet-induced obesity^[18]^. This hallmark study was followed by many reports showing the beneficial effects of *A. muciniphila* administration in a variety of mouse models^[19-23]^. However, all these studies have been using mucin-based media to cultivate *A. muciniphila*, providing a potential bias since this animal-derived glycoprotein is not free from remnants of other bacteria. A breakthrough came with the development of metabolic models that showed the dependency of *A. muciniphila* on exogenously added threonine and its inability to synthesize N-acetylglucosamine (GlcNAc) from glucose, resulting in synthetic media that obviated the use of mucin by using a combination of L-threonine, glucose, GlcNAc, and peptone^[24,25]^. These were used in a series of mechanistic studies in diabetic and obese mice, demonstrating that pasteurized *A. muciniphila* cells replicated the beneficial effects of live cells grown in mucin-free media in diabetic and obese mice^[26]^. The capacity of pasteurized *A. muciniphila* cells to be at least as effective as live cells was confirmed in a recent clinical trial where their administration to metabolic syndrome subjects resulted in improved insulin sensitivity, reduced insulinemia and plasma total cholesterol, and reduction of body weight, including reduced fat mass and hip circumference^[27]^.

Several studies have been focusing on the cultivation, storage, and delivery methods of either pasteurized or alive *A. muciniphila* for therapeutic purposes^[26,28-31]^. Moreover, the environmental conditions in which *A. muciniphila* survives have been studied in detail, as it is sensitive to low pH, oxygen and bile salts^[12,32,33]^. This information may be useful for the delivery of live *A. muciniphila* for therapeutic purposes. Along with the development of synthetic media, the growth characteristics and the physiology of *A. muciniphila* have been characterized by comparing mucin growth to that in media containing single sugars, such as glucose and GlcNAc. This is important as *A. muciniphila* exerts its health benefits while using mucin as a carbon, nitrogen, and energy source, and hence, several studies addressed its transcriptome and proteome under these conditions^[24,32]^. Recent years have seen an increasing number of signaling molecules that *A. muciniphila* is producing, which interact with the host^[1,14]^. These include the protein Amuc_1100, which is known to be part of a set of outer membrane proteins^[34]^. Preclinical data have shown that the heat-stable Amuc_1100 protein can reproduce the effects of live and pasteurized cells in protection from diet-induced obesity and is an efficient ligand for signal transduction to Toll-like receptor 2 (TLR2)^[26,35]^. Other recently identified proteins that have been implicated in host signaling include Amuc_1631 (also known as P9), Amuc_1434, and Amuc_2109, but the location and production of these have not yet been studied^[36-39]^.

For human interventions or supplementation with *A. muciniphila* cells, industrial-scale fermentations are needed and thus the used cultivation media should be not only free from mucin derived from animals but also food-grade, non-allergenic and enable efficient growth to high densities to provide cost-effective production platforms. Moreover, to address present consumer needs, such as increasing interest in flexitarian diets and sustainability, plant-based rather than animal-derived components are to be used. Finally, in these conditions, there should be sufficient production of signaling molecules that have been identified as interacting with the host. It is furthermore of importance to assess the safety of *A. muciniphila* in the development trajectory for its use in therapeutic applications. A recent study demonstrated the safety of pasteurized *A. muciniphila* cells in a variety of in vitro models and a 90-day rat trial^[40]^. This and other information was used by the European Food Safety Authority (EFSA) to approve the use of pasteurized *A. muciniphila* cells as a novel food^[41]^. This all supports the interest in the fermentation optimization of this next-generation beneficial microbe, and in this study, we assessed the growth and performance of *A. muciniphila* in newly developed food-grade and plant-based media with varying carbon sources using a multi-omics approach in comparison with its growth on mucin-containing media^[42]^.

## METHODS

### Bacterial strain and culture conditions

The type-strain *A. muciniphila* Muc^T^ (ATCC BAA-835) was used for all cultivation experiments.

Basal medium was used in the fermentations for the initial experiments including soy medium and mucin medium and pre-cultures for the pea peptone bioreactors as described previously^[12,43]^.

For the initial experiments, soy medium and mucin medium were prepared. To prepare soy medium, 16 g/L soy peptone (AM41, Organotechnie SAS) was added to basal medium. In addition, GlcNAc and glucose were added in equimolar amounts to a total of 25 mM (Sigma-Aldrich). Mucin medium was prepared by adding 0.5% hog gastric mucin (Sigma-Aldrich) to basal medium.

Pre-cultures grown for the anaerobic fermentations supplemented with pea peptone were cultivated in basal medium supplemented with tryptone (20 g/L) and L-threonine (4 g/L) with the following carbon source composition: 12.5 mM GlcNAc and 12.5 mM glucose. The cultures were grown in anaerobic conditions and incubated at 37 °C for 48 h (non-shaking).

Food-grade medium was used for the main experiments with the following composition: KH_2_PO_4_ (0.4 g/L), Na_2_HPO_4_ (0.669 g/L), NH_4_Cl (0.3 g/L), NaCl (0.3 g/L), MgCl_2_ 6H_2_O (0.1 g/L), pea peptone A482 (OrganoTechnie SAS, 32 g/L), and L-threonine (4 g/L). After autoclaving 2 mL of reducing solution containing NaHCO_3_ (40 g/L) and L-cysteine^*^HCl (5 g/L) and 1% (v/v) of vitamin solution (see above) were added to the medium. The medium was inoculated with 1% (v/v) of the pre-culture. Anaerobic fermentations using this medium were performed as described in the next section.

### Anaerobic fermentation

The fermentations were conducted in four parallel bioreactors (DasGip, Eppendorf, Germany) using 700 mL of food-grade medium containing either glucose and GlcNAc in three different ratios (3:1, 10:1 or 20:1 glucose to GlcNAc, named A, B, and C, respectively) or mucus (Condition D) with a final total concentration of 150 mM or 0.5% crude mucin. The pH was set at 6.8 and a stirring rate of 100 rpm was applied with N_2_/CO_2_ gas flow (80%/20%). The medium of all four bioreactors was inoculated with 1% (v/v) of cultures pre-grown on tryptone medium. The fermentation was terminated after 72 h. Samples were taken for optical density (OD) measurements, high performance liquid chromatography (HPLC) analysis, microscopic analysis, RNA sequencing (triplicates), and proteomics (duplicates). Samples for HPLC analysis were stored at -20 °C until use. Samples (10 mL) for RNA sequencing and proteomics were taken at early, mid, and end exponential phases and centrifuged for 30 min at 4,700 rpm at 4 °C, after which the supernatant was removed. Then, 1 mL of RNAlater was added to the pellets of the samples for RNA sequencing. Lastly, the samples for both RNA sequencing (in triplicate) and proteomics (in duplicate) were snap-frozen in liquid nitrogen and stored at -80 °C.

### HPLC

Samples were obtained at different time points during the fermentation period for the analysis of fermentation products. Crotonate was used as the internal standard. The external standards were GlcNAc, glucose, acetate, propionate, succinate, lactate, and 1,2-propanediol. The substrates and fermentation products were measured using a Shimadzu LC_2030C equipped with a refractive index detector and a Shodex SH1011 column. Two runs were performed for each sample, employing oven temperatures of 45 and 75 °C, with pump flow rates of 1.0 and 0.9 mL/min, respectively. For both runs, 0.01N H_2_SO_4_ was used as eluent. All samples and standards (10 µL) ran for 15 min. The concentrations of the standards were ranging between 2.5 and 60 mM. Lastly, the fermentation profiles obtained with HPLC were used to calculate the carbon and energy balances at the endpoint of all fermentations.

### RNA isolation and transcriptome analysis

RNA isolation was performed as described previously^[44]^. Further processing of the total RNA was performed by Novogene (Cambridge, United Kingdom) and paired-end sequences of 150 bp were obtained using an Illumina platform. Transcriptome analysis has been performed as previously described^[33]^. All further analysis was done using R version 3.6.3 in Rstudio version 1.2.5019.

## RESULTS

### Growth characteristics and metabolic activity

In the first series of experiments, we built on the metabolic modeling data that predicted *A. muciniphila* Muc^T^ to grow efficiently (growth rate of 0.13 h^-1^) on an equimolar mixture of glucose and GlcNAc in a minimal medium with threonine^[24,25]^. To increase cell yield, a food-grade and plant-based protein source was added to the minimal medium in the form of 16 g/L soy protein hydrolysate that resulted in a medium (soy medium) yielding a high growth rate of 0.53 h^-1^ exceeding that of *A. muciniphila* on mucin, which is approximately 0.41 h^-1^^[12,35]^. The cell densities in the soy peptone medium, as measured by absorption at OD600, were above 5, whereas the mucin medium supported growth to an OD of 2-2.5^[24]^. Even higher cell yields could be obtained by using pea peptone at a level of 32 g/L, which led to high densities of OD600 values above 10. Since the soy protein hydrolysate is derived from a plant source, it is an acceptable food-grade nitrogen source and applicable on a large scale. Moreover, as the growth rate on soy medium was higher than that on mucin and relatively high cell densities were obtained, we decided to further characterize *A. muciniphila* cells grown on this food-grade medium, the more so as these were highly active in protecting mice from diet-induced obesity^[26]^. A few observations were noted in the first series of experiments that needed to be further addressed. First, phase-contrast and scanning electron microscopy of cells grown on soy medium showed a significantly (*P*-value < 0.01) elongated shape with a length of 1.3 (±0.80) µm *vs.* 0.8 (±0.25) µm when grown in mucin medium (based on analysis of 251 and 160 cells, respectively) [Supplementary Figure 1]. Additionally, the acetate/propionate ratio in the soy medium was 0.92, while that of cells grown in mucin was 1.2 (±0.13)^[32]^. This can be explained by the fact that the GlcNAc, which is present in equal amounts as glucose in the soy medium, generates an extra acetate after deamination, as reported previously^[24,25]^.

To further address the global differences between *A. muciniphila* cells grown in soy medium and mucin medium, transcriptome analysis was performed to reveal initial transcriptional changes between mucin and soy medium (Supplementary Table 1 and see below).

The main differences were found to be in the increased expression in soy medium of genes encoding transporters such as Major Facilitator Superfamily (MFS), biopolymer, anion and amino acid transporters (Amuc_1331, Amuc_0546, Amuc_0221 and Amuc_0037), as well as peptide, aliphatic sulfonate, nitrate/sulfonate/bicarbonate, cobalt and manganese ABC transporters (Amuc_0672, AMUC_1297, Amuc_0408, Amuc_1198, Amuc_1199, Amuc_0056, Amuc_1380 and Amuc_1186) with an increase > 5-fold. In addition, genes involved in oxygen stress response were found to be higher in soy medium including rubrerythrin (Amuc_2055 and Amuc_2056), peroxidase (Amuc_1321), and catalase (Amuc_2070). In mucin medium, genes involved in cell shape (Amuc_0540) and division (Amuc_0348) and mucin degradation genes such as alpha-N-acetylglucosaminidase (Amuc_0060), beta-glucanase (Amuc_0875), and sulfatases (Amuc_0491 and Amuc_0451) were found to have an > 5-fold increase.

### High biomass yield reached on food-grade medium

**Figure 1:**
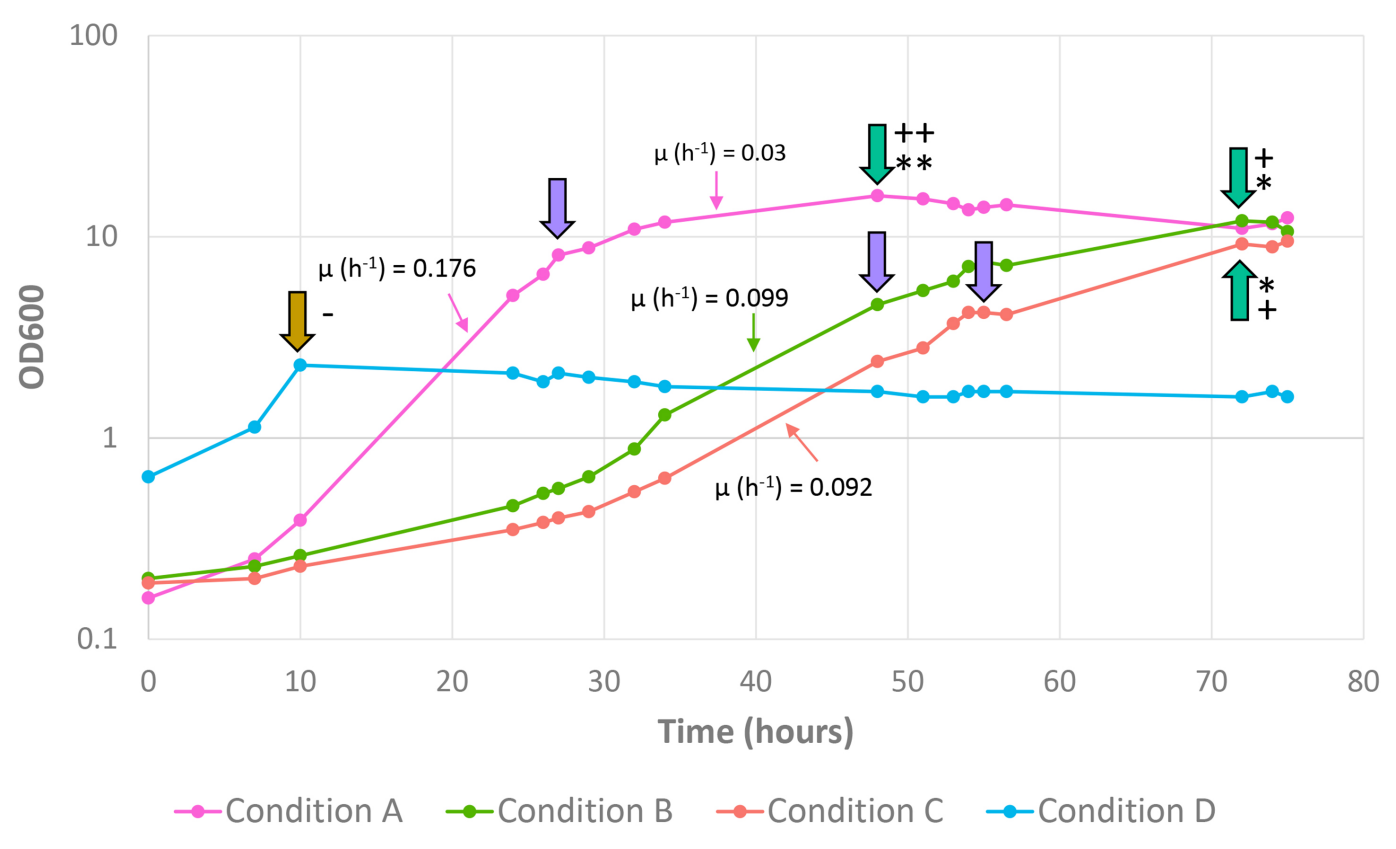
Exponential growth of *A. muciniphila* as a function of glucose to GlcNAc ratios. Bioreactors at pH 7.0 were run with glucose to GlcNAc carbon source ratios of 3:1 (Condition A), 10:1 (Condition B), and 20:1 (Condition C) or on mucin (Condition D). Purple arrows indicate mid-log sampling, turquoise arrows indicate end-of-growth sampling, and the brown arrow indicates sampling of condition D. The growth rate is indicated in the graph for conditions A-C. Asterisks at the arrows indicate either glucose and GlcNAc were depleted (^**^) or only GlcNAc was depleted (^*^). Observed viscosity in the cultures is indicated with (++) meaning high viscosity, (+) meaning medium viscosity, or (-) no viscosity observed. *A. muciniphila*: *Akkermansia muciniphila*; GlcNAc: N-acetylglucosamine.

Because of the apparent effect of equimolar amounts of glucose and GlcNAc on the morphology, viscosity, and gene expression of *A. muciniphila* cells, we further explored the effect of different carbon source ratios on the growth and physiology of *A. muciniphila*. For this purpose, we decided to use pea peptone as an additional food-grade nitrogen source rather than soy peptone to avoid potential issues associated with phytoestrogens present in soy. A total of four fermentations were characterized in detail, with three different glucose to GlcNAc carbon source ratios 3:1 (Condition A), 10:1 (Condition B), and 20:1 (Condition C) and one control, which was supplemented with mucin (Condition D). Interestingly, while the mucin medium enabled a rapid initiation of growth, an increase in the duration of the lag phase was observed along with the decreasing concentrations of GlcNAc in the bioreactors [Figure 1]. It is important to note that the pre-cultures were grown on pea peptone medium supplemented with equimolar concentrations of glucose and GlcNAc, which is most similar to condition A in terms of glucose to GlcNAc ratio. However, up to four transfers in food-grade medium supplemented with glucose and GlcNAc in a ratio of 20:1 were found to lead to growth adaptation of *A. muciniphila* and rapid initiation of growth [Supplementary Figure 2]. Microscopy results showed the formation of elongated cells in conditions A-C as compared to mucin (condition D) [Supplementary Figure 3]. The number of cells and increased cell length observed in these cultures reflect a higher biomass production. Condition A was found to have the fastest growth and highest biomass density, as deduced from the OD600 measurements.

### Transcriptome response in exponential and stationary phase

**Figure 2:**
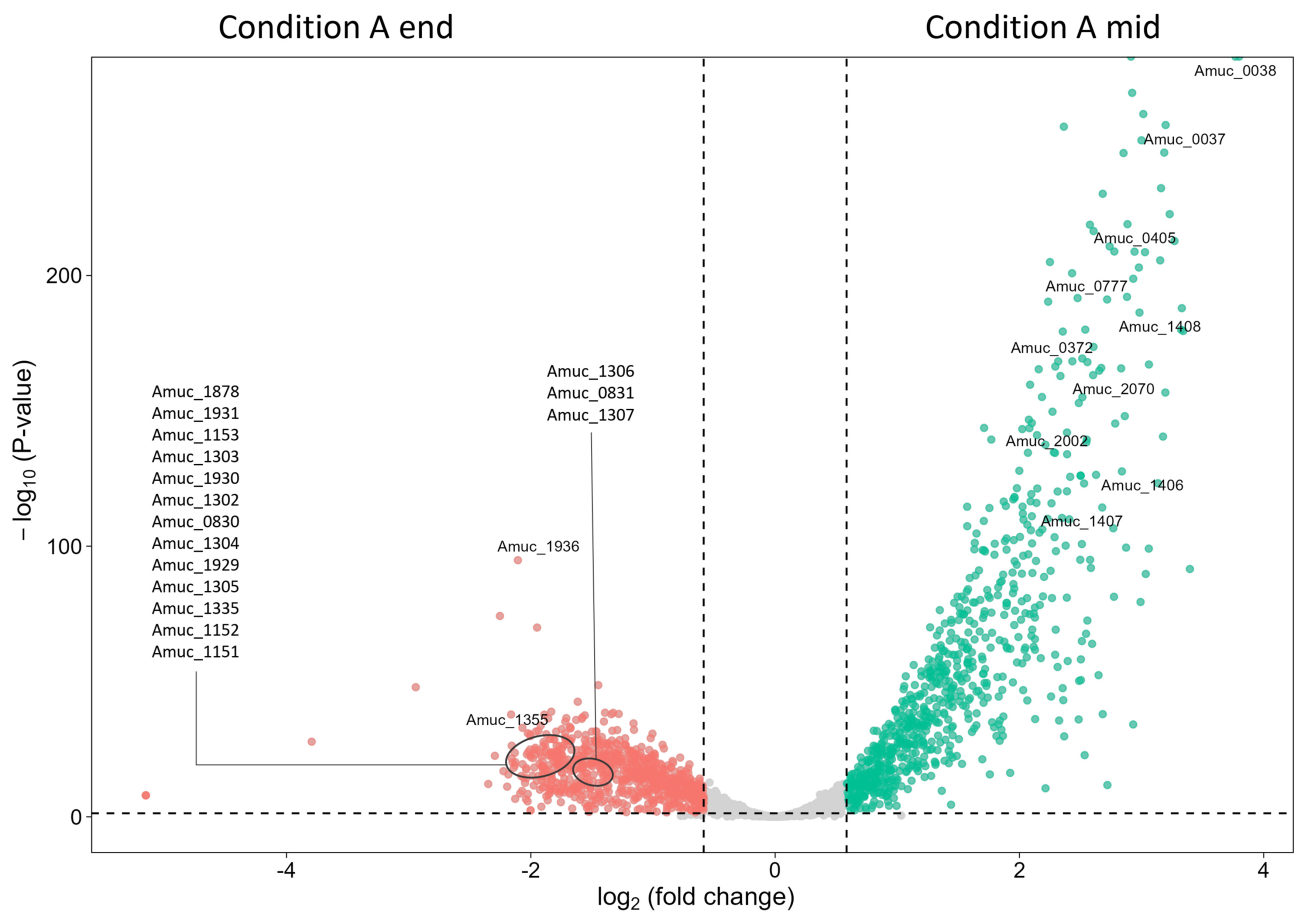
Volcano plot comparing the transcriptional response of *A. muciniphila* in fermentations of the mid-log phase and end-growth phase of condition A. Only the differentially expressed genes mentioned in the text are labeled here. The complete overview of differentially expressed genes and their annotation can be found in Supplementary File 1. *A. muciniphila*: *Akkermansia muciniphila.*

The growth curve of condition A showed two different growing phases, the first one until approximately 27 h with a high growth rate (0.18 h^-1^) and the second phase from 27 to 48 h (0.03 h^-1^), the time at which the glucose and GlcNAc were depleted [Figure 1]. This observation was also supported by the transcriptome data that showed dozens of genes to be significantly higher expressed in the first compared to the second growth phase [Figure 2, Supplementary Table 2, Supplementary Figure 4 and Supplementary File 1]. The genes with the highest upregulation (> 5-fold) at the end of the first phase included several gene clusters with two or more juxtaposed genes, such as the metabolic gene cluster encoding a glutaminase and a likely glutamine-GABA antiporter (Amuc_0037-0038), as well as several stress proteins (Amuc_1406-1408, coding for DnaK, GroES and GroEL). In addition, the expression of single genes was upregulated for other stress proteins such as Skp (Amuc_0405) and HtpG (Amuc_2002), as well as genes involved in oxygen and other stress responses, such as catalase (Amuc_2070), glutamate decarboxylase (Amuc_0372), and NAD(P)-dependent oxidoreductase (Amuc_0777). Many of the genes observed to be upregulated in the end-growth phase of this condition had no known function and were annotated as hypothetical. Genes that were upregulated and annotated with a function included genes involved in transport systems for iron (Amuc_1929-1931), potassium (Amuc_0830-0831 and Amuc_1151-1153), and phosphate (Amuc_1302-1307), as well as phage production (such as Amuc_1355, Amuc_1936, and Amuc_1335). A notable exception was the highly (14.8-fold) upregulated gene for a predicted lactoylglutathione lyase (Amuc_1878) that has shown in *Salmonella* to be involved in the detoxification of methylglyoxal, known to be produced in the gut but also in bioreactors with peptones^[45]^. The transcriptome data are also supported by proteome data, which showed that the global proteome is more conserved than the global transcriptome during different growth conditions [Supplementary File 2, Supplementary Figures 5 and 6].

In addition to the transcriptional activity, the catabolism of *A. muciniphila* in the food-grade medium was assessed to determine the depletion of the glucose and GlcNAc [Figure 1 and Supplementary Figure 7]. In all conditions, the main fermentation products included acetate, propionate, and succinate. As shown previously, the propionate:acetate ratio may differ depending on the cultivation conditions used to grow *A. muciniphila*^[25]^. In the conditions tested in this study, the ratio of propionate:acetate varied between the different carbon source ratios used for cultivation [Supplementary Figure 8]. Comparing the final stages of the bioreactors, the lowest propionate:acetate ratio was found in condition D with a ratio of 0.88, followed by condition A with a ratio of 1.06, while the ratios in condition B and C were 1.25 and 1.23, respectively, coinciding with the ratios found in the initial experiments on soy medium. The carbon balances of the fermentations with glucose and various amounts of GlcNAc were calculated and amounted to approximately 72% [Supplementary Figure 9].

### GlcNAc concentration affects the expression of glycosyltransferases and stress response genes

**Figure 3:**
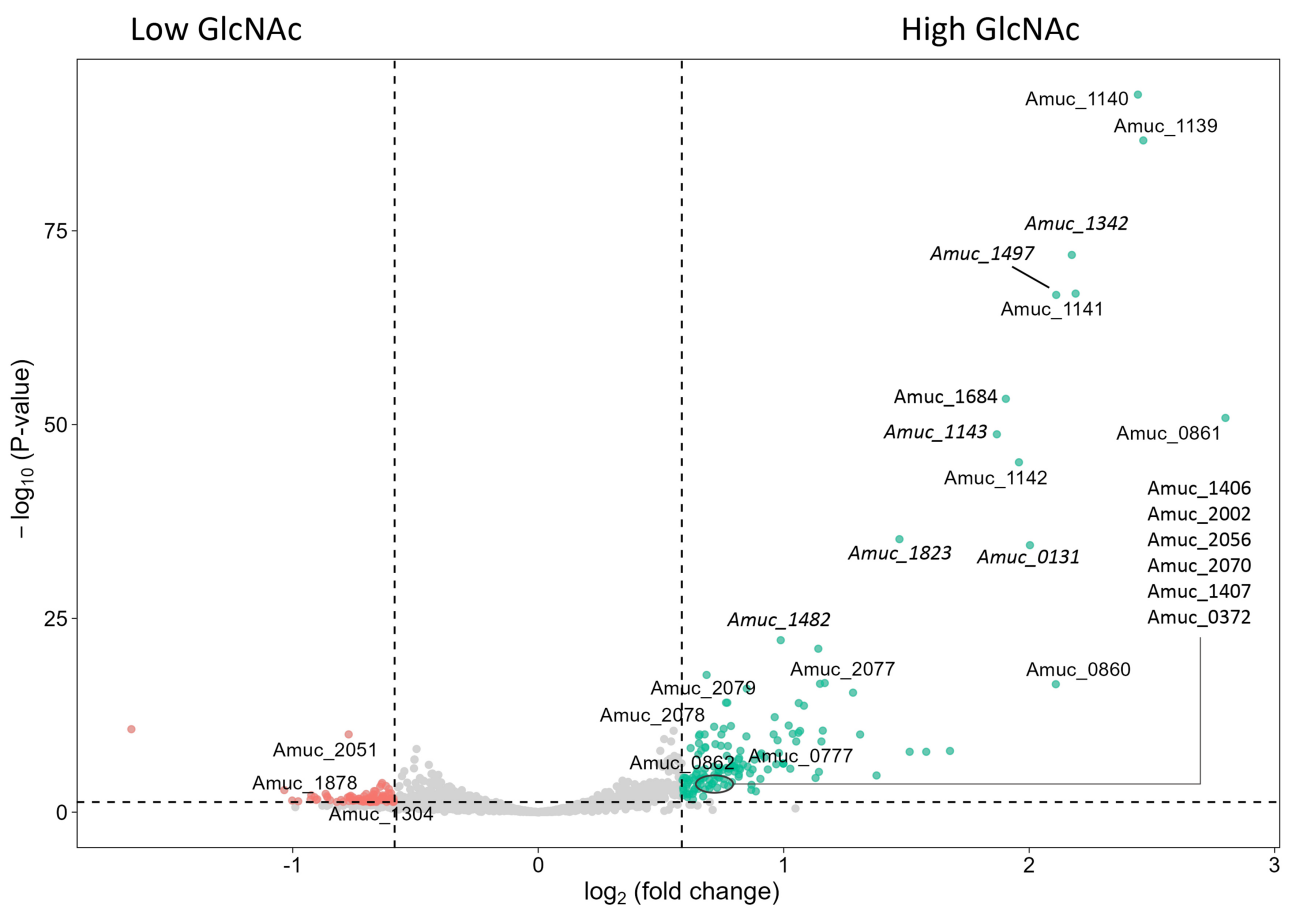
Volcano plot comparing the transcriptional response of *A. muciniphila* in fermentations containing a high concentration of GlcNAc (mid-log condition A) and a low concentration of GlcNAc (mid-log condition C). The differentially expressed genes mentioned in the text are labeled here, as well as highly differentiated genes annotated as hypothetical proteins in italics. The complete overview of differentially expressed genes and their annotation can be found in Supplementary File 1. *A. muciniphila*: *Akkermansia muciniphila*; GlcNAc: N-acetylglucosamine.

A limited number of genes were found to be differentially expressed between the mid-log phases of conditions with different GlcNAc concentrations. Due to the small number of differentially expressed genes between the mid-log phases of conditions B and C (two significant differentially expressed genes, Amuc_1139 and Amuc_1140, both of which belong to a glycosyltransferase cluster), here we only compare condition A (high GlcNAc) and condition C (low GlcNAc) (252 differentially expressed genes) [Figure 3].

A higher concentration of GlcNAc showed significantly higher expression of a glycosyltransferase cluster (Amuc_1139 until Amuc_1142) and genes possibly involved in exopolysaccharide or capsular polysaccharide production (Amuc_2077 until Amuc_2079). Furthermore, a higher concentration of GlcNAc led to overexpression of genes involved in stress response, including an anaerobic ribonucleoside triphosphate reductase cluster (Amuc_0860 until Amuc_0862), NAD(P)-dependent oxidoreductase (Amuc_0777), rubrerythrin (Amuc_2056), catalase (Amuc_2070), glutamate decarboxylase (Amuc_0372), and molecular chaperones DnaK (Amuc_1406), HtpG (Amuc_2002), and GroES (Amuc_1407). In low GlcNAc, many genes encoding hypothetical proteins were found among the upregulated genes. However, lactoylglutathione lyase (Amuc_1878), glutamate dehydrogenase (Amuc_2051), and phosphate ABC transporter permease protein PstA (Amuc_1304) were significantly upregulated in this condition as compared to high GlcNAc.

### Proteomic and transcriptomic response of *A. muciniphila* in glucose/GlcNAc *vs.* mucin

Cultivating *A. muciniphila* in food-grade medium where mucin is substituted for a mixture of glucose and GlcNAc may induce transcriptional and translational changes that affect its physiology. Using the proteome data, we compared mid-log condition A (high GlcNAc) and condition D (mucin). In total, 116 proteins were identified to have a protein abundance ratio higher than 10. The protein abundance ratios indicate that in the mucin condition, most proteins involved in mucin degradation were upregulated [Supplementary Table 3]. In contrast, in high GlcNAc medium, the proteins that were upregulated in comparison to mucin were mainly stress-related proteins and glycosyltransferases, in line with the observations related to the transcriptional response.

In the following sections, we focus mainly on the transcriptome data since these revealed a higher number of differentially expressed genes than that found in the proteomics data [Supplementary Table 2]. This suggests that cells responded to differences in medium composition by adapting its regulations over its functional aspect, possibly minimizing differences in overall cell composition. In the following sections, we compared the transcriptional response of exponentially growing cells (mid-log) on high GlcNAc or low GlcNAc and mucin.

### High GlcNAc induces stress response as compared to mucin

**Figure 4:**
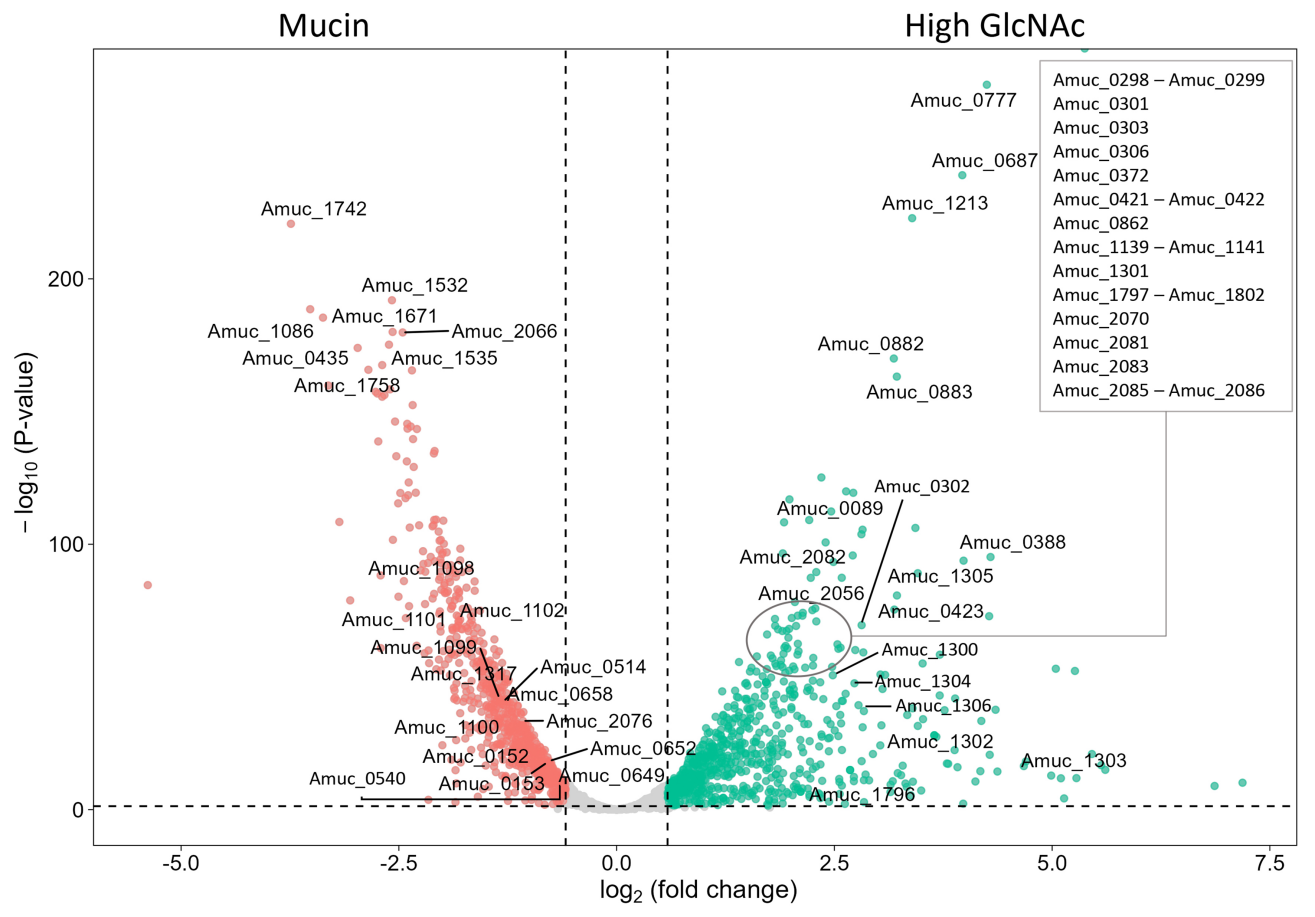
Volcano plot comparing the transcriptional response of *A. muciniphila* in fermentations containing a high concentration of GlcNAc (mid-log condition A) and mucin (condition D). Due to the high number of genes upregulated in high-GlcNAc, genes with a -log10 *P*-value < 1 × 10^-50^ and a log2 fold change < 2.5 that are mentioned in the text are not shown in this figure. All other differentially expressed genes mentioned in the text are labeled here. The complete overview of differentially expressed genes and their annotation can be found in Supplementary File 1. *A. muciniphila*: *Akkermansia muciniphila*; GlcNAc: N-acetylglucosamine.

In the medium containing high GlcNAc (Condition A), several genes and complete gene clusters related to stress responses were found to be significantly upregulated as compared to the mucin condition [Figure 4]. This includes a gene cluster encoding for an ABC transporter and phosphate ABC transporter (Amuc_1294-1308), a gene cluster involved in the production of exopolysaccharides (Amuc_2077-Amuc_2096), and an additional significantly upregulated glycosyltransferase cluster (Amuc_1139-1142), a gene cluster containing multiple aldo/keto reductases (Amuc_1796-1809), and the gene for anaerobic ribonucleoside-triphosphate reductase activating protein (Amuc_0860). Moreover, additional complete gene clusters related to stress responses were found to be significantly upregulated in both high-GlcNAc and low-GlcNAc conditions, as compared to mucin, including a gene cluster encoding ribosomal proteins (Amuc_0294-0308), an iron transport cluster (Amuc_1930 until Amuc_1934), and a potential flavin biosynthesis gene cluster (Amuc_0421-0426) [Supplementary File 1]. Other genes that may be involved in stress response but not part of a gene cluster were also identified to be upregulated in both GlcNAc conditions. This includes genes for rubrerythrin (Amuc_2055-2056), catalase (Amuc_2070), oxidoreductases (Amuc_0116, Amuc_0777, Amuc_1072, Amuc_1176 and Amuc_1389), ribonucleoside-triphosphate reductase (Amuc_0862), and glutamate decarboxylase (Amuc_0372).

Next to the transcriptomic stress response, elongated cells were observed in the bioreactors containing glucose and GlcNAc in comparison to the small oval-shaped cells visible when *A. muciniphila* was cultivated on mucin [Supplementary Figure 3]. Elongated cells were also observed in the previously mentioned soy medium cultures [Supplementary Figure 1], together with an increased expression of Amuc_0540 encoding cell shape-determining protein MreB in mucin as compared to soy medium. Following this observation, we performed a more in-depth analysis of the expression of genes involved in cell elongation and division [Table 1 and Supplementary File 1]. Overall, the transcriptomic stress response and elongated cells observed in the cultures grown in synthetic medium without mucin indicate that a stress response is triggered in the absence of mucin in this medium.

**Table 1:** Differential expression of genes involved in cell division comparing high-GlcNAc to the mucin condition

**Genes**	**Description**	**High-GlcNAc *vs.* mucin**		
		**Fold change**	***P* value**	
Amuc_1176	Cell division inhibitor	2.62	6.57E-48	Upregulated in high GlcNAc
Amuc_0348	Cell division protein FtsH	1.50	4.32E-36	
Amuc_1052	Cell division trigger factor	1.43	7.41E-31	
Amuc_1558	RIP metalloprotease RseP	0.22	1.63E-06	
Amuc_0662	Polypeptide-transport-associated domain-containing protein FtsQ-type	0.31	4.58E-08	Upregulated in mucin
Amuc_0540	Cell shape-determining protein MreB^*^	0.45	6.6E-11	
Amuc_0652	Peptidoglycan glycosyltransferase	0.59	4.79E-19	
Amuc_0649	Transcriptional regulator MraZ	0.62	3.34E-17	
Amuc_0153	Cell division protein FtsA	0.64	1.97E-18	
Amuc_0152	Tubulin/FtsZ GTPase	0.88	1.24E-26	
Amuc_2076	Cell division FtsK	1.16	3.66E-34	
Amuc_0658	Cell cycle protein	1.50	3.38E-41	
Amuc_0514	Peptidoglycan glycosyltransferase	1.67	1.26E-41	
Amuc_1317	Integral membrane protein CcmA involved in cell shape determination	2.03	2.88E-46	

^*^Genes that were also upregulated in the preliminary transcriptome data comparing soy medium to mucin medium. GlcNAc: N-acetylglucosamine; RIP: regulated intramembrane proteolysis.

## DISCUSSION

In this study, we assessed the cultivation of *A. muciniphila* food-grade pea-peptone medium with different concentrations of carbon sources to produce cells suitable for therapeutic applications. The use of food-grade synthetic and non-allergenic medium supplemented with glucose and GlcNAc resulted in high cell yields and fast growth of *A. muciniphila*. Furthermore, we gained detailed insight into physiology by a combination of biochemical analysis as well as transcriptional and proteomic analysis. In addition, we compared the use of the food-grade synthetic medium to the mucin medium, which has been used in many studies to grow *A. muciniphila* cells for animal studies^[18-23]^.

The highest growth rate and final optical density were reached in food-grade medium containing the highest concentration of GlcNAc. In the GlcNAc conditions, the cells were observed to be elongated, possibly affecting the optical density in these cultivations. Furthermore, PCA analysis showed that in both the proteome and transcriptome data, the mucin condition clusters separately from the conditions containing GlcNAc, whereas in the transcriptome data alone, the end-growth phase of condition A also clusters separately from the other conditions and time points. However, the KEGG metabolism on general level 1 did not reveal significant differences between fermentor conditions within the different metabolisms. It may be possible that the regulation of several genes evened out the impact and differences would be visible at a deeper level. Assessing the transcriptome and proteome data in more detail revealed the upregulation of proteins and genes involved in stress response in GlcNAc conditions and the pili-associated system, mucin degradation, and protein sorting systems in mucin conditions.

A shift in propionate to acetate production was observed between the condition containing mucin and the condition containing glucose and GlcNAc, as well as between the different glucose and GlcNAc conditions. At the end of the fermentation in mucin, a propionate:acetate ratio of 0.88 was observed. However, with the decreasing concentration of GlcNAc in the other conditions, the ratio shifted toward more propionate production. In condition A, the ratio was 1:1, which was similar to previous findings, where a 50:50 ratio of glucose and GlcNAc was used as a carbon source^[25]^. The shift toward a higher propionate:acetate ratio in conditions B and C is in line with the degradation reactions that were predicted using the genome-scale model of *A. muciniphila*^[24]^. Therefore, it is important to note that using a lower GlcNAc concentration in the cultivation of *A. muciniphila* causes a shift in the propionate:acetate ratio, resulting in an altered short-chain fatty acid profile.

The carbon recovery values indicated a gap between the carbon sources that were consumed and the energy and carbon sources that were produced. The carbon recovery values ranged between 70%-73%, excluding biomass and amino acid formation. Previously, a carbon recovery of 80%-90% has been described for *A. muciniphila* cultivated using either GlcNAc, glucose, or N-Acetylgalactosamine (GalNAc) as carbon sources^[24]^. Due to the high amount of pea peptone in this medium, the exact biomass could not be measured. Therefore, we hypothesize that by including a theoretical portion for biomass, our carbon recoveries may be in the range of the previously observed carbon recoveries for *A. muciniphila*. In addition, considering the elongated cells and the production of exopolysaccharides (EPS), as our transcriptome data indicate, a portion of the initial carbon concentration available in the medium may be used for cell wall and EPS production. Next to the transcriptome data, the observed viscosity in the cultures containing GlcNAc and glucose suggests EPS may be produced in these conditions.

Cell elongation was observed in the fermentations without mucin. As described previously, the cells of *A. muciniphila*, when cultivated on mucin medium, are oval-shaped and approximately 0.6 μm in diameter and 0.7 μm in length^[12]^. In synthetic medium, the cells were elongated, sometimes 2-3 times [Supplementary Figure 1] and compared to mucin, several genes involved in cell division were found to be upregulated. Three genes, namely cell division inhibitor (Amuc_1176), FtsH (Amuc_0348), and the cell division trigger factor (Amuc_1052), were significantly upregulated under high and low GlcNAc conditions. FtsH encodes a metalloprotease, which plays a role in the quality control of integral membrane proteins in *E. coli*. In *A. muciniphila*, this gene may be upregulated due to the elongated membranes that were observed, increasing the quality control of these membrane proteins. Furthermore, the overproduction of the trigger factor was found to cause defective cell division in *E. coli*^[46]^. Therefore, the upregulation of this gene Amuc_1052 may also be involved in the observed cell elongation of *A. muciniphila*. The upregulation of cell division genes in the mucin condition supports the differences observed in cell size between mucin and glucose/GlcNAc conditions.

The protein Amuc_1100 is gaining increasing interest in several studies for its positive effect on host health^[35,47]^. The gene cluster associated with pili production, including Amuc_1100, showed significant upregulation in the condition supplemented with mucin compared to the GlcNAc conditions. The proteome data support this observation, but the protein abundance ratio observed was less than 10. However, as the proteome data sets relate to relative amounts of proteins, absolute amounts of proteins could be higher in the GlcNAc conditions as the OD600 value was approximately 8-fold higher in the high-GlcNAc condition than the mucus condition.

The upregulation of multiple stress-related genes and gene clusters was identified in fermentations on GlcNAc compared to mucin. First, the upregulation of the phosphate ABC transporter system may indicate a phosphate limitation in these cultures^[48]^. To overcome this limitation, additional phosphate sources could be added to the food-grade medium. Furthermore, several genes previously found to be involved in the oxygen stress response of *A. muciniphila* were found to be upregulated in conditions without mucin as well^[32]^. However, these fermentations were run anaerobically simultaneously, as was the case with the mucin condition. Therefore, it is likely that this stress response was not specific for oxygen, but rather a form of cross-protection against other stresses^[49]^. Another stress response that was activated in conditions containing glucose and GlcNAc was the production of EPS (Amuc_2077-2096). Interestingly, the expression of the genes involved in EPS production decreased along with the decreasing concentration of GlcNAc, as well as the glycosyltransferase cluster (Amuc_1139 until Amuc_1142). The production of EPS in bacteria is often a mechanism to cope with harsh environmental conditions, as extensively studied for lactic acid bacteria^[50]^. For *A. muciniphila*, the high-GlcNAc condition, with limiting medium components, may be sub-optimal. Additionally, within condition A, it was visible that during the mid-growth phase, stress-related genes were upregulated as compared to the end phase; this included metabolic gene cluster encoding a glutaminase and a likely glutamine-GABA antiporter (Amuc_0037-0038), as well as several stress proteins (Amuc_1406-1408, coding for DnaK, GroES and GroEL). However, the upregulation of stress-related genes does not limit growth rate and biomass production as observed in these fermentations. We showed that pea peptone-based food-grade medium supplemented with glucose and GlcNAc instead of mucin results in high biomass formation of *A. muciniphila*, which may be used for its production for therapeutic purposes.

Other comparisons of the growth of *A. muciniphila* on mucin versus specific carbon sources have been made^[24,25]^. A transcriptome comparison was conducted between cultivations supplemented with mucin and those supplemented with glucose^[24]^. Despite the differences in cultivation, cultures with glucose as a carbon source also showed upregulation of stress-related genes in comparison to mucin^[24]^. When comparing the data of the previous study^[24]^ and the present one, we noted that the expression of many genes was similar. Furthermore, similar differences were also documented for genes and proteins involved in mucin degradation in both studies. In the present proteome analysis, we found that on the protein level, it was clearly visible that the mucin culture had an upregulation of proteins involved in mucin degradation, since these proteins were located in the top protein abundance ratios. Similar findings were documented previously^[24]^. In contrast, there were also differences between the previous study^[24]^ and the present one, notably including differentially expressed stress-related genes. Moreover, the differences noted here in the expression of genes involved in cell division were not found in the earlier study. This may be due to the low growth rates and final biomass that were observed in the cultures with mucin only, while in our studies with synthetic media, high growth rates and biomass were observed.

In conclusion, we showed that pea peptone-based food-grade medium supplemented with glucose and GlcNAc instead of mucin results in high biomass formation of *A. muciniphila*, which may be used for its production for therapeutic purposes. The differences between growth on mucin and growth on glucose and GlcNAc were shown at transcriptome and proteome levels. However, the general level 1 sKEGG metabolism pathways showed high similarity between conditions and no major significant differences could be identified. Furthermore, if minimal EPS production is required, the transcriptome data indicate that this may be achieved by decreasing the GlcNAc concentration. Lastly, the use of synthetic medium affects the cell morphology of *A. muciniphila*, resulting in elongated cells. Overall, our data suggest that the food-grade synthetic medium composition described here could be used to produce *A. muciniphila* in high yields for therapeutic purposes. This has recently been shown in the first proof-of-concept study with *A.muciniphila* Muc^T^ grown on such a synthetic medium that showed the capacity of live and pasteurized cells to improve several metabolic parameters in overweight and obese volunteers^[24]^.

## DECLARATIONS

### Availability of data and materials

Data supporting our findings are published as Supplementary Materials in this journal.

### Copyright

© The Author(s) 2024.
