# Supplementary material for: Omics-based analysis of Akkermansia muciniphila cultivation in food-grade media
Source: Microbiome Res Rep. 2024 Jun 17;3(3):36. doi: 10.20517/mrr.2024.06 (PMC11480725; doi:10.20517/mrr.2024.06)
Supplement: Supplementary file 1 [file mrr-3-3-36-SupplementaryMaterials.zip › mrr3006-SupplementaryFigures.pdf]

Supplementary Figures

Omics-based analysis of *Akkermansia muciniphila* cultivation in food-grade media

Sharon Y. Geerlings[1], Kees van der Ark[1], Bart Nijsse[2], Sjef Boeren[3], Mark van Loosdrecht[4], Clara Belzer[1], Willem M. de Vos[1,5]

[1]Laboratory of Microbiology, Wageningen University, Wageningen 6708 WE, the Netherlands.
[2]Laboratory of Systems and Synthetic Biology, Wageningen University, Wageningen 6708 WE, the Netherlands.
[3]Laboratory of Biochemistry, Wageningen University, Wageningen 6708 WE, the Netherlands.
[4]Department of Biotechnology, Delft University of Technology, Delft 2629 HZ, the Netherlands.
[5]Human Microbiome Research Program, Faculty of Medicine, University of Helsinki, Helsinki 00014, Finland.

Correspondence to: Prof. Willem M. de Vos, Laboratory of Microbiology, Wageningen University, Helix building, Stippeneng 4, Wageningen 6708 WE, the Netherlands. E-mail: willem.devos@wur.nl

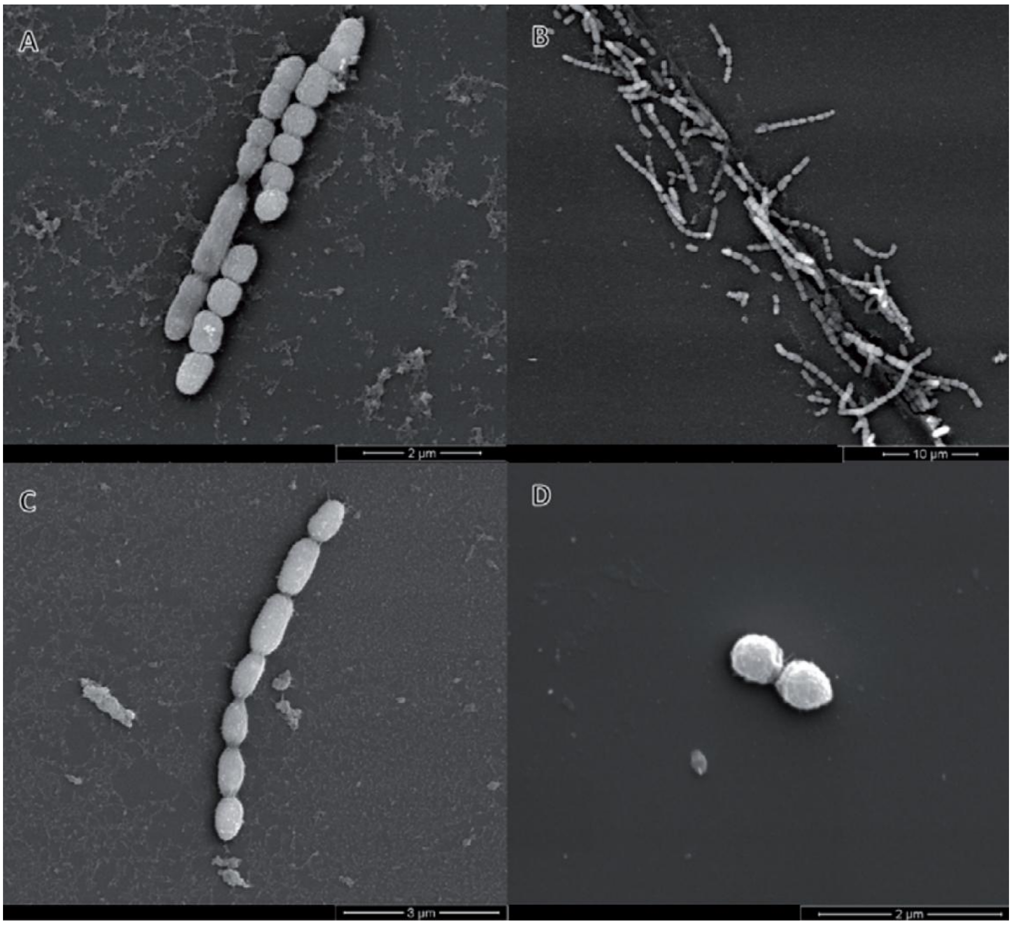

Supplementary Figure 1. Scanning electron micrographs of *A. muciniphila* cultured on soy medium (A, B and C). The cells are elongated or do not divide properly. Normal-shaped cells were observed in mucus medium (D).

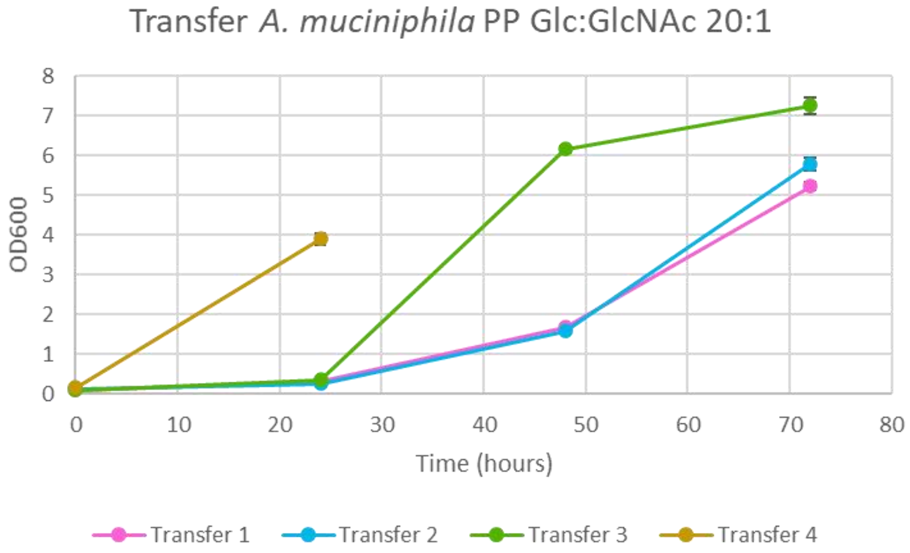

Supplementary Figure 2. OD measurements of *A. muciniphila* after multiple transfers to a new serum bottle using the medium composition of condition C.

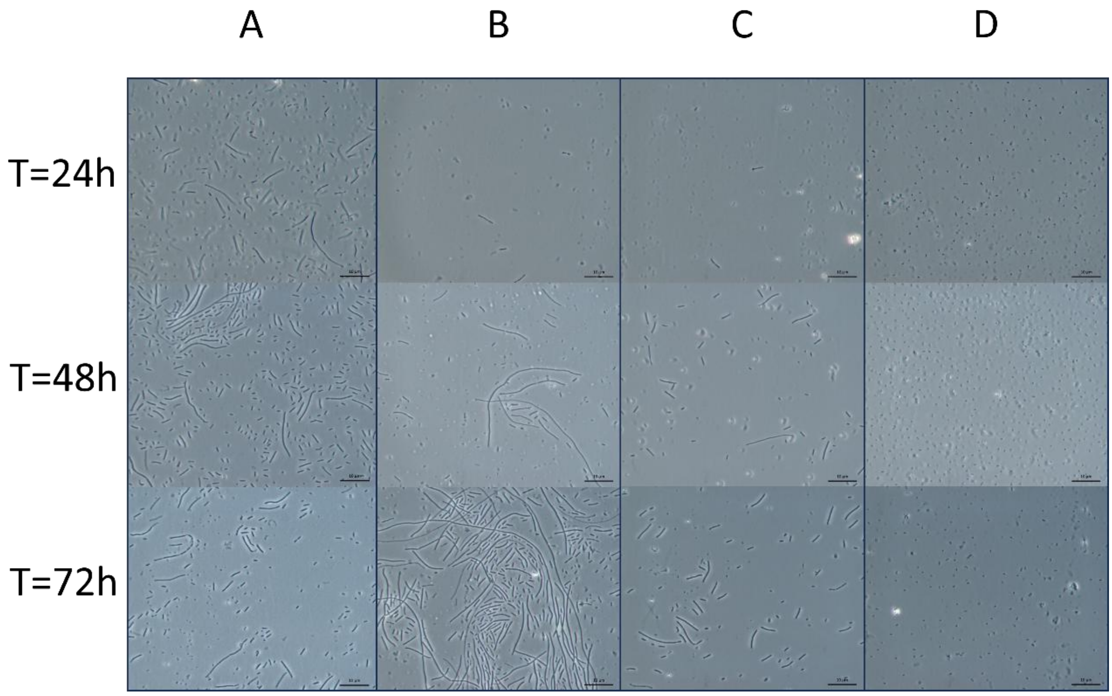

Supplementary Figure 3. Microscope images of all conditions over time.

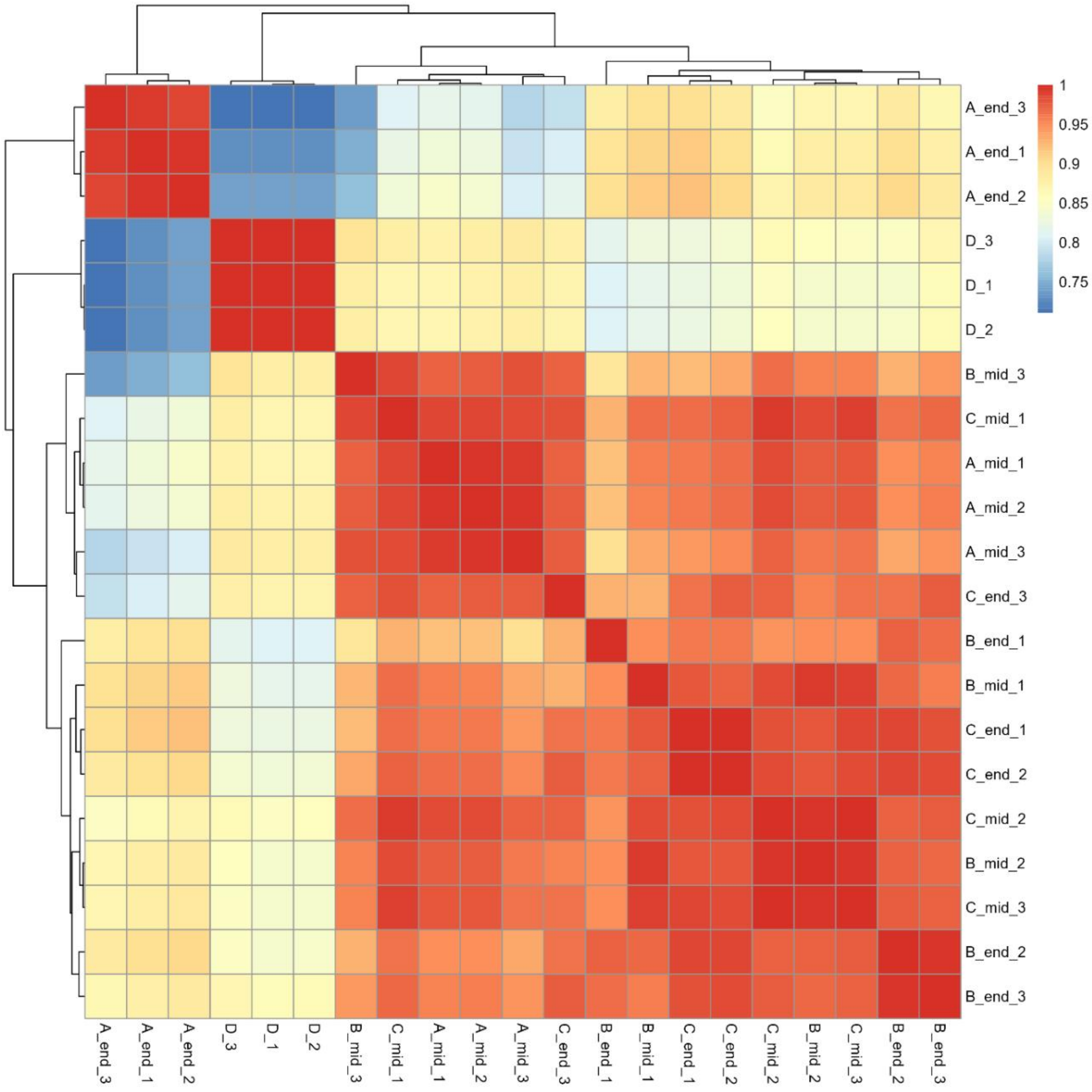

Supplementary Figure 4. Heatmap based on the transcriptome data of all samples.

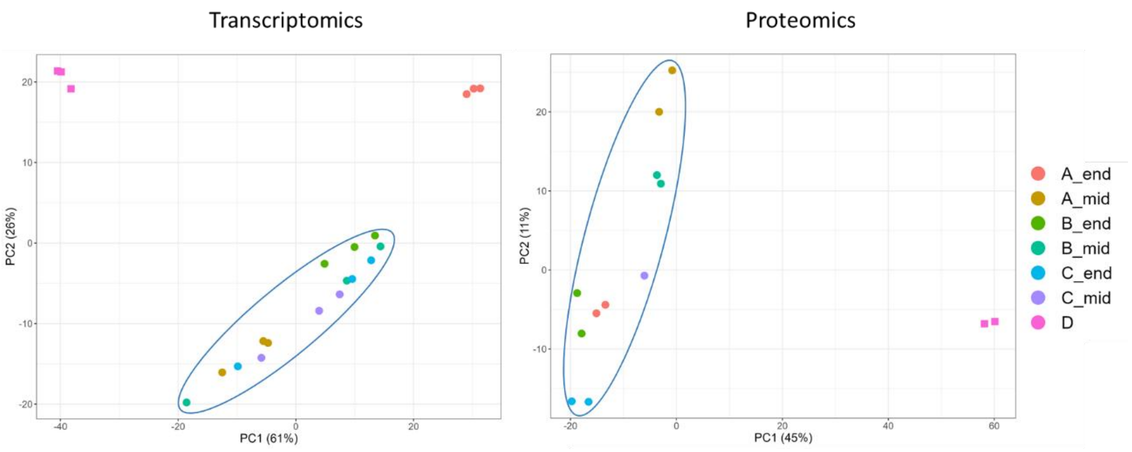

Supplementary Figure 5. PCA analysis of fermentor conditions A-D over time, for transcriptomics n=3 and proteomics n=2. The main clusters are highlighted for clarity; squares represent the results with mucin-grown cells. The PCA based on the proteome data did not show separation between A_end and the other samples, indicating that on the proteome level, these samples are more similar than observed on the transcriptome level.

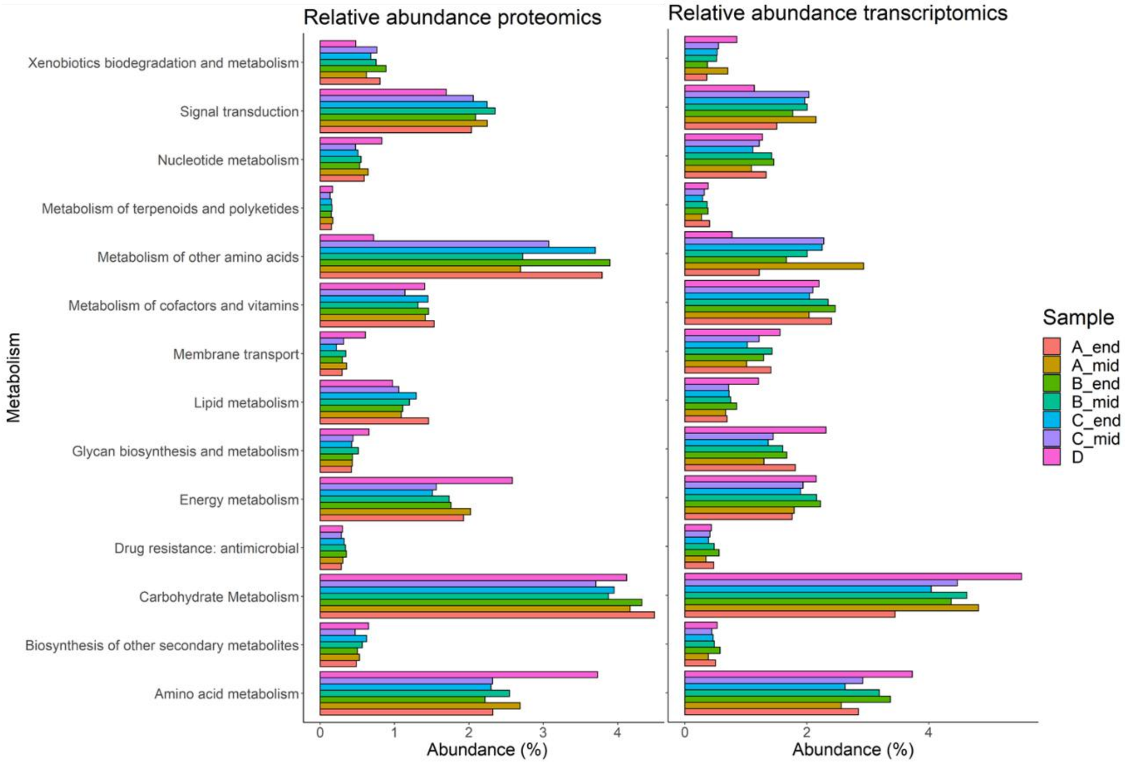

Supplementary Figure 6. KEGG metabolism in proteome and transcriptome data across different conditions. Metabolism of other amino acids includes the following pathways: beta-alanine, D-glutamine, D-glutamate, glutathione, phosphonate, phosphinate, and selenocompound metabolism. KEGG metabolic pathway predictions were used to assess the differences in the metabolic pathways operating in various fermentor conditions (Figure S8). Overall, the metabolic pathway with the highest abundance across all conditions was found to be the carbohydrate metabolism pathway, with abundances ranging between 3.4% and 5.5% for transcriptomics and between 3.7% and 4.4% for proteomics. The metabolism pathway with the second highest abundance at the transcriptome level was the amino acid metabolism pathway, with abundances ranging from 2.6% to 3.7%. However, an exception was noted for the mid-log phase of condition A, where the metabolism of other amino acids has the second highest abundance of 2.93%. In contrast, the proteome showed a higher abundance of proteins involved in the metabolism of other amino acids (2.7%-3.9%) as opposed to the findings in the transcriptomics data. Apart from condition D, where the abundance of proteins involved in the metabolism of other amino acids was only 0.72%, statistical analysis revealed no significant differences between fermentor conditions within the different metabolisms.

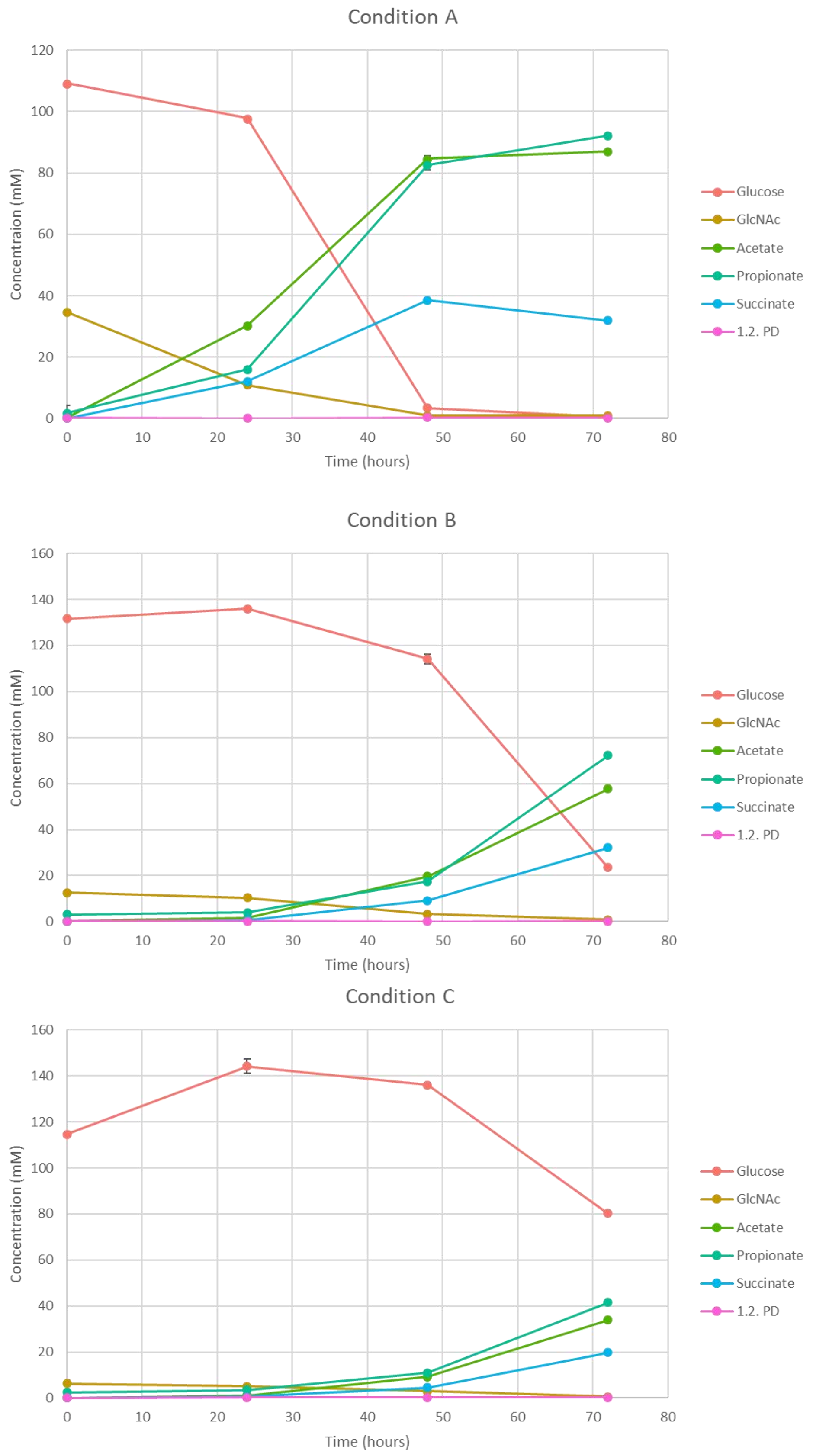

Supplementary Figure 7. Sugar uptake and metabolite production by *A. muciniphila* on cultivation conditions A, B and C.

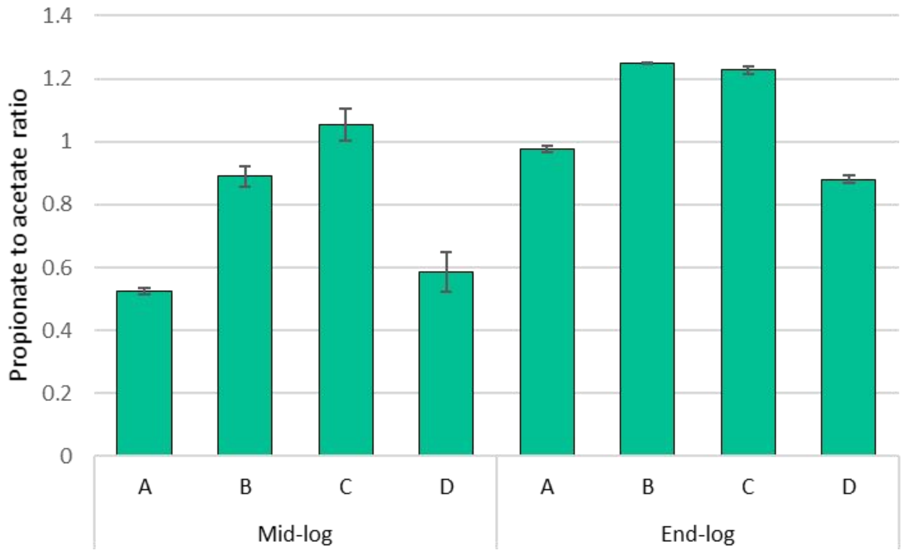

Supplementary Figure 8. Overview of propionate:acetate ratios over time.

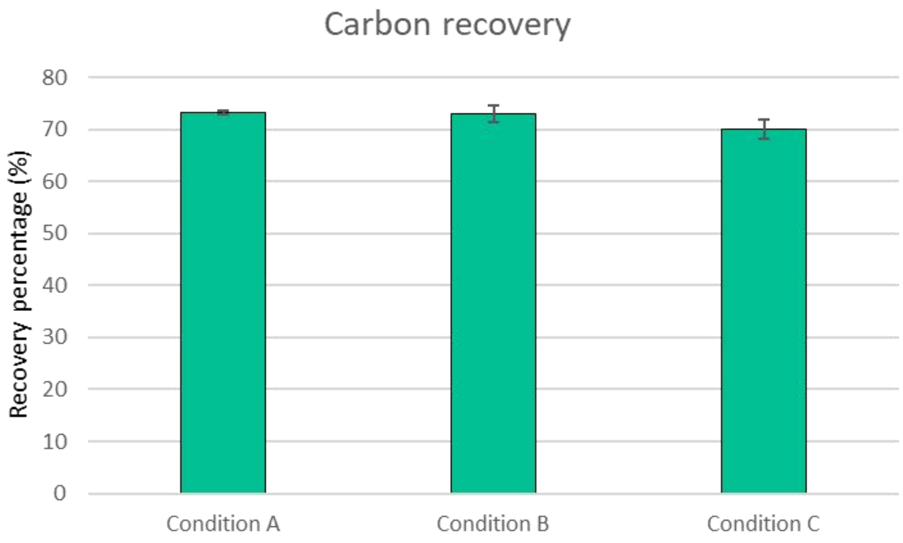

Supplementary Figure 9. Electron and carbon balances of condition A-C calculated at the endpoint of each fermentation.
