# Supplementary material for: Omics-based analysis of Akkermansia muciniphila cultivation in food-grade media
Source: Microbiome Res Rep. 2024 Jun 17;3(3):36. doi: 10.20517/mrr.2024.06 (PMC11480725; doi:10.20517/mrr.2024.06)
Supplement: Supplementary file 1 [file mrr-3-3-36-SupplementaryMaterials.zip › mrr3006-SupplementaryFile2.pdf]

Sample preparation for nLC-MS/MS and analysis

Total protein was isolated from culture samples. Ice-cold 100 mM Tris buffer (pH 8) was added to the pellets in variable amounts up to approximately 500 µl and 200 µl of the resuspended cells was transferred to low-binding Eppendorf tubes. The tubes were centrifuged repeatedly for 1 min at 10.000 x g until the supernatant was clear. Then, the supernatant was removed and 200 µl of ice-cold 100 mM Tris buffer (pH 8) was added again. The cells were resuspended after which the centrifugation was repeated. After removing the last supernatant, the cells were resuspended in 200 µl 100 mM Tris (pH 8) and aliquoted per 50 µl in low-binding Eppendorf tubes. Subsequently, the samples were sonicated for 15 seconds. The protein concentration was measured using the Pierce™ BCA Protein Assay Kit to dilute all samples to a concentration of 1 µg/µl in a final volume of 60 µl using 100 mM Tris. Then, 6 µl of 150 mM dithiothreitol (Sigma Life Science) was added to the diluted sample followed by incubation at 37°C for 45 minutes. After incubation, the sample was mixed with 198µl 8 M urea (Sigma-Aldrich) and 27 µl 150 mM acrylamide and incubated for 30 minutes at room temperature. Subsequently, 4 µl of 10% trifluoroacetic acid (TFA, Alfa Aesar Chemicals) and 8 µl SpeedBeadsTM magnetic carboxylate modified particles (50%, GE Healthcare 45152105050250 and 50% Thermo Scientific 65152105050250, washed twice with MilliQ water) and 750 µl acetonitrile (Biosolve B.V.) were added to the sample solution. Next, the mixtures were incubated for 20 minutes at room temperature and the liquid was removed. Then, the beads were washed twice on a magnetic rack (Cell Signaling Technology) with 1 mL 70% ethanol and with 1 mL 100% acetonitrile, respectively. Afterwards, 100 µl of 5 ng/µl trypsin (Roche Diagnostic GmbH) in 50 mM ammonium bicarbonate was added to the beads containing the sample and subjected to overnight digestion at room temperature. The digestion was stopped by adding 4 µl 10% TFA to the sample. Then, the samples were placed on the magnetic rack to obtain the first supernatant. The remaining beads were washed with 100 ml/L formic acid. This solution was then combined with the first supernatant. Micro Columns (µcolumns) were prepared by adding two C18 disks (Affinisep AttractSPE™ Disk Bio C18), 200 µl methanol and 4 µL 50% Lichroprep RP-18 in methanol into a 200 µL pipette tip. The µcolumn was then eluted and washed with 100 µl methanol and equilibrated with 100 µl 1 mL/L formic acid in water before use. The samples with the cell lysates were then transferred to the µcolumn. After elution and washing with 100 µL 1 mL/L formic acid in water, 50 µl of (1:1) formic acid and acetonitrile solution was added onto the µcolumn and eluted into a clean 0.5 mL low-binding Eppendorf tube. Subsequently, the samples were concentrated to a final volume of approximately 10 µl using an Eppendorf Concentrator Plus.

Lastly, the volume was adjusted to 50 µl using 1 mL/L formic acid before storage at -20°C until further processing.

Peptide samples were measured (nLC1000 - Orbitrap Exploris 480) and data analysis was performed as previously described [1]. The database that was used for the analysis of the samples in this study was the Akkermansia_muciniphila_baa-835_UP000001031 uniprot database.

References:

1. Feng Y, Bui TPN, Stams AJM, et al. Comparative genomics and proteomics of Eubacterium maltosivorans: functional identification of trimethylamine methyltransferases and bacterial microcompartments in a human intestinal bacterium with a versatile lifestyle. *Environ Microbiol* 202210.1111/1462-2920.15886.10.1111/1462-2920.15886.
